# A PCBM Electron Transport Layer Containing Small Amounts of Dual Polymer Additives that Enables Enhanced Perovskite Solar Cell Performance

**DOI:** 10.1002/advs.201500353

**Published:** 2015-12-10

**Authors:** Zonglong Zhu, Qifan Xue, Hexiang He, Kui Jiang, Zhicheng Hu, Yang Bai, Teng Zhang, Shuang Xiao, Kenan Gundogdu, Bhoj Raj Gautam, Harald Ade, Fei Huang, Kam Sing Wong, Hin‐Lap Yip, Shihe Yang, He Yan

**Affiliations:** ^1^ Department of Chemistry and Energy InstituteThe Hong Kong University of Science and TechnologyClear Water BayKowloonHong Kong; ^2^ Institute of Optoelectronic Materials and DevicesState Key Laboratory of Luminescent Materials and DevicesSouth China University of TechnologyGuangzhouP. R. China510641; ^3^ Department of PhysicsThe Hong Kong University of Science and TechnologyClear Water BayKowloonHong Kong; ^4^ Department of PhysicsNorth Carolina State UniversityRaleighNCUSA27695

**Keywords:** doping of electron transport layer, highly efficient inverted perovskite solar cells, negligible hysteresis

## Abstract

**A polymer/PCBM hybrid electron transport layer** is reported that enables high‐performance perovskite solar cells with a high power conversion efficiency of 16.2% and with negligible hysteresis. Unlike previous approaches of reducing hysteresis by thermal annealing or fullerene passivation, the success of our approach can be mainly attributed to the doping of the PCBM layer using an insulating polymer (polystyrene) and an amine‐containing polymeric semiconductor named PFNOX.

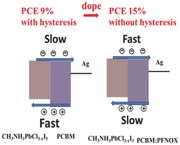

This is an open access article under the terms of the Creative Commons Attribution License, which permits use, distribution and reproduction in any medium, provided the original work is properly cited.

The field of organic–inorganic metal halide perovskite solar cells (PKSCs) has seen impressive progresses in recent years.^1^ The rapidly increasing power conversion efficiency (PCE) of PKSCs has attracted much attention of both the academic and industrial research communities and makes PKSCs one of the most promising candidates for the next generation of large scale cost‐competitive photovoltaic technology.^2^ The attractive features of PKSCs include high absorption characteristics, appropriate direct band gaps, high carrier mobility, long charge carrier diffusion length, low cost, and easy fabrication processes.^3^ These merits had pushed the PCE of PKSCs to a record value of 20.1% recently.^4^ The conventional PKSC structure^5^ consists of an electron transport layer (ETL) (e.g., TiO_2_) with the perovskite layer on top and then capped with a hole transport layer (HTL, such as spiro‐OMeTAD). In the past two years, however, inverted structure PKSCs (with a typical structure of indium tin oxide (ITO)/poly(3,4‐ethylenedioxythiophene) polystyrene sulfonate (PEDOT:PSS)/perovskite layer/phenyl‐C_61_‐butyric acid methyl ester (PCBM)/top electrode) have developed rapidly and can now achieve comparable PCEs with the conventional structure PKSCs.^6^

One of the main challenges for PKSCs is that typical PKSCs exhibit large hysteresis in their *J–V* characteristics, which makes PKSCs difficult to characterize or to produce in a reproducible and repeatable manner.[[qv: 2c]],^7^ The hysteresis problem is typically less severe for conventional structure PKSCs based on the mesoporous TiO_2_. This is because the mesoscopic TiO_2_ offers a large surface area and the charges generated in the perovskite layer can be transported to the TiO_2_ mesoporous film or via the self‐connected perovskite network.^8^ In contrast, it is more challenging to optimize the interfaces in planar‐structure inverted PKSCs and to achieve a perfectly balanced electron and hole currents in the device. Thus far, several reports have shown hysteresis‐less PKSCs by long‐time thermal annealing, filling with PCBM,^9^ and by balancing the electron and hole transports in the perovskite cell.^10^ However, the majority of the reports on inverted PKSCs are still troubled with various extents of the hysteresis problem and there are limited reports of high‐efficiency inverted PKSCs with completely eliminated hysteresis.^11^ Indicated by previous reports,[[qv: 6e]],[[qv: 11a]] the hysteresis problem could be related to the hole and electron transport layers (e.g., need to achieve balanced charge transport)[[qv: 6e]] as well as the perovskite active layer (e.g., need to grow large crystals and eliminate traps in the perovskite active layer).[[qv: 11a]] Therefore, the complete elimination of hysteresis in PKSCs may require the combination of different approaches that address different components of the perovskite cells. As most previous work has been focused more on the perovskite active layer than the PCBM ETL, we hope to improve the PCBM ETL to provide a simple and effective approach to complement previous ones.

In our previous work, we have developed a series of polyfluorene‐based semiconducting polymers that contain alkylamine side chains.[[qv: 11b]],^12^ One of the classical materials in the family is PFNOX, which has been widely used as interfacial layers for organic light emitting diodes, field effect transistors, and polymer solar cells.^13^ In our recent work on polymer solar cells, it was discovered that PFNOX can electrically dope PCBM and thus enables efficient electron transfer between the active layer and the interlayer of the polymer solar cell devices.^12^, ^14^ The excellent electron transfer characteristics and effective electrical doping of PFNOX to PCBM for polymer solar cells inspired us to use PFNOX as a polymer additive to electrically dope the PCBM ETL for PKSCs. It is expected that improved electron transport property of the PCBM upon PFNOX doping should benefit the performance of PKSCs.

In this paper, we report an improved PCBM ETL that contains ≈95% of PCBM and two polymer additives, namely, PFNOX and polystyrene (PS), at very small weight percentages (1%–3%). As been demonstrated in our previous report,^15^ the function of the polystyrene (with a high molecular weight of 10 m kDa) additive is mainly to increase the viscosity of the ETL solution and thus improving the quality of the ETL films. The high film quality of the ETL also helps to suppress undesirable electron recombination caused by the defects in the ETL, which contributes to the increased *V*_oc_ of the cell. In this paper, discussions will be mainly focused on the dramatic impacts of using PFNOX as the electrical dopant of the PCBM ETL. When neat PCBM or PCBM/PS are used as the ETL, the PKSCs exhibit large hysteresis and can only achieve PCE of ≈11%. With the additional PFNOX dopant, however, the PKSCs based on PCBM/PFNOX or PCBM/PFNOX/PS as the ETL exhibit dramatic improvements on cell performance with their PCEs increased to 14% and 16.2%, respectively. Most importantly and surprisingly, the cells based on ETL containing the PFNOX dopant exhibits dramatically reduced hysteresis. Our best cell with PCBM/(PFNOX&PS) ETL shows higher efficiency and negligible hysteresis, compared to the devices‐based neat PCBM ETL. Combining the study of the transient photoluminescence (PL) and electrochemical impedance spectroscopy (EIS), it was found that the PFNOX doped PCBM ETL facilitates the electron injection from halide perovskite into the ETL and decreases the recombination rate of charge carriers. Our hybrid polymer/PCBM ETL offers a simple yet highly effective approach to improve the performance and reduce the hysteresis of PKSCs. The fundamental studies on the doped ETL also offer important insights on understanding the hysteresis problem and charge transfer and transport processes in PKSCs.

The representative perovskite solar cell device with the structure of ITO/PEDOT:PSS/perovskite/PCBM:PFNOX/Ag is shown in **Figure** **1**A. The chemical structure of PFNOX and PCBM are shown in Figure 1B. PFNOX is one of the best‐performance example of the alkyl‐amine‐containing polyfluorene conjugated polymer family. In a typical PFNOX/PCBM hybrid ETL, there is only a small amount of (1.25 wt%) PFNOX in the PCBM layer. A PFNOX solution (5 mg mL^−1^) was added into a 20 mg mL^−1^ PCBM solution at a volume ratio of 1:20 and stirred overnight at 60 °C. The energy levels of the PCBM:PFNOX hybrid ETL are similar to those of the neat PCBM films, which are confirmed by the similar peaks in the UV–vis absorption spectra (Figure S1, Supporting Information) and cyclic voltammograms (Figure S2, Supporting Information) of the corresponding films. Figure 1C presents the typical cross‐sectional scanning electron microscopy (SEM) image of a high performance photovoltaic device. A well‐crystallized mixed halide perovskite (CH_3_NH_3_PbCl_3−*x*_I*_x_*) layer with a thickness of 450 nm is sandwiched between the PEDOT‐PSS HTL and the PCBM: PFNOX ETL. Thus, a total thickness of the cell is ≈1 μm including a 200–300 nm ITO layer. To prove that the chlorine is homogeneously distributed in the perovskite layer, we show the SEM mapping, the enerdy dispersive X‐ray spectroscopy (EDS) picture and element distribution (Figure S4 and Table S3, Supporting Information) of the perovskite layer. The data show that the chlorine ions are homogeneous and the chlorine only has small percentage in the MAPbCl_3−*x*_I*_x_*, which is in agreement with previous reports.^15^

**Figure 1:**
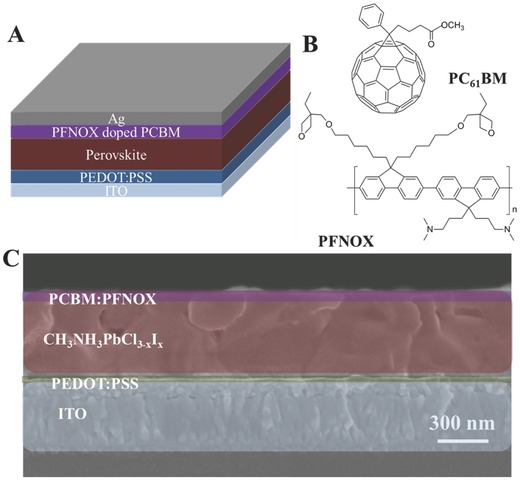
A) Illustration of typical perovskite solar cells with a structure of ITO/PEDOT:PSS/perovskite/PCBM:PFNOX/Ag and B) the molecular structure of PCBM and PFNOX. C) Cross sectional SEM image of the perovskite solar cell.

The photocurrent density–voltage (*J–V*) characteristics measured under AM 1.5G irradiation at a one sun intensity of 100 mW cm^−2^ for cells with and without PFNOX additives are shown in **Figure** **2**. The detailed performance parameters of these devices are summarized in **Table** **1**. A typical device using the pure PCBM as the ETL can only achieve a PCE of ≈11.4%, with a short‐circuit photocurrent (*J*_sc_) 18.6 mA cm^−2^, an open‐circuit voltage (*V*_oc_) of 0.952 and a fill factor (FF) of 0.668. However, when loading the PFNOX additive into the PCBM layer, the best performance has increased 14.0% with the *V*_oc_ increasing to 0.94 V, *J*_sc_ increasing to 20.4 mA cm^−2^, and FF increasing to 0.729. This efficiency increase can be mainly attributed to the increases of the *J*_sc_ and FF. The incident photon‐to‐electron conversion efficiency (IPCE) spectrum is displayed in Figure 2B showing a remarkable increase of the integrated IPCE area upon PFNOX doping.

**Table 1:** Photovoltaic performance parameters of solar cells based on different PCBM composite electron transport layers with different scanning direction

ETL/devices	Scan direction	*V*_oc_ [V]	*J*_sc_ [mA cm^−2^]	FF [%]	PCE [%]	PCE average [%]
PCBM	Forward	0.92	18.6	66.8	11.4	10.6
	Reverse	0.92	18.3	58.3	9.8	
PCBM:PS	Forward	0.98	18.5	65.2	11.8	11.1
	Reverse	0.98	18.4	58.7	10.5	
PCBM:PFNOX	Forward	0.94	20.4	72.9	14.0	13.7
	Reverse	0.94	20.5	69.4	13.4	
PCBM:(PFNOX&PS)	Forward	1.01	20.8	76.9	16.2	16.0
	Reverse	1.01	20.8	74.5	15.7	

**Figure 2:**
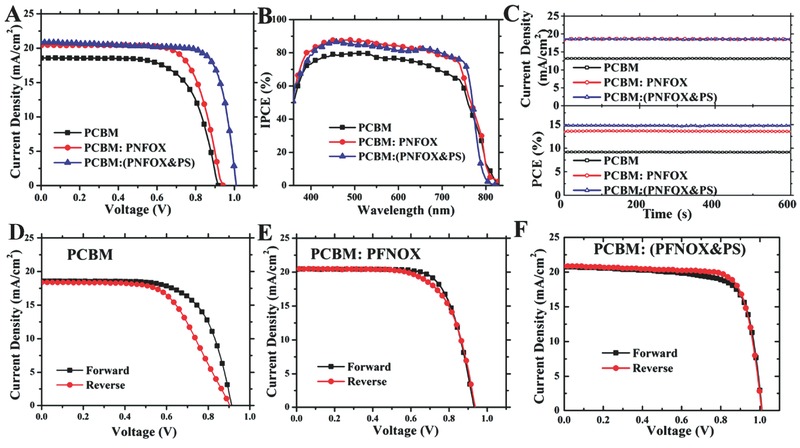
A) Current density–voltage (*J–V*) curves and B) incident photo‐to‐electron conversion efficiency (IPCE) of perovskite solar cell based on PCBM, PCBM:PFNOX, and PCBM: (PFNOX&PS) electron transport layer. C) The stabilized photocurrent density (up) and PCE (down) obtained while holding the solar cell near the maximum power point voltage at 0.69 V for PCBM, 0.73 V for PCBM:PFNOX, and 0.84 V for PCBM: (PFNOX&PS), respectively. *J–V* curves for with D) and E) without PFNOX additives and with F) PS&PFNOX additives which measured by forward (from short circuit to open circuit) and reverse (from open circuit to short circuit) scans. All *J–V* curves were measured under 100 mW cm^−2^ air mass 1.5 global (AM 1.5G) illumination.

In order to improve the uniformity of the PCBM ETL and further enhance the PCE performance, a second polymer additive, polystyrene (*M*_w_ ≈ 10 m), was included into the PCBM/PFNOX ETL. As shown in our previous report, the PS additive can increase the viscosity of the solution, thus improve the uniformity of the PCBM ETL.^15^, ^16^ As also shown in Figure 2A, the PS additive further increased the PCE of the PKSCs to an impressive value of 16.2%, with a *V*_oc_ of 1.01 V, a *J*_sc_ of 20.8 mA cm^−2^, and a FF of 0.769. To check whether the high PCE of our devices is reliable, the stabilized photocurrent was measured at a photovoltage near the maximum power point of the optimal device. The photocurrent was then monitored and plotted as a function of time. As shown in Figure 2C, we obtained a steady‐state PCE of 13.5% for PCBM:PFNOX‐based device and 15.6% for that based on PCBM:(PFNOX&PS), thereby which is in agreement with the average PCE of the related devices. However, the PCBM only based device has a low PCE of 9.4%. For comparison, we also fabricated devices using the standard structure (FTO/TiO_2_/MAPbCl_3−*x*_I*_x_*/Spiro‐OMeTAD/Ag) and obtained an efficiency of 14.5% (Table S5, Supporting Information).

Hysteresis has been one of major problems for PKSCs, which could be attributed to grain boundaries and defects that cause strong recombination between the perovskite and charge transport layer.^17^ Several groups previously reported less‐hysteresis effect for inverted planar heterojunction PKSCs with a structure of PEDOT:PSS/perovskite/PCBM.^9^, ^18^ One of the proposed reasons for the reduced hysteresis was the thermal passivation of the perovskite layer after long‐term high temperature annealing, during which small molecular PCBM deposited onto the trap states of perovskite surface and eliminate the photocurrent hysteresis.^1^ Surface passivation is another approach to decrease the surface defects and reduce the recombination in the perovskite film.[[qv: 18b,c]] In previous studies,[[qv: 8a]],^19^ the fundamental causes of hysteresis have been attributed to several possible factors including grain boundaries in the perovskite films and charge‐trapping defects at the perovskite and transport layer interfaces. Previous approaches were mainly focusing on improving the quality of perovskite layer by removing defects or growing large crystals. In contrast, our study focused on improving the PCBM electron transport layer. Figure 2D–F presents the three *I*–*V* curves for pure PCBM, PCBM:PFNOX, and PCBM: (PFNOX&PS) ETLs‐based photovoltaic devices. For the PFNOX and PS codoped PCBM devices, they both show *J–V* curves with negligible hysteresis, with PCEs of 16.2% and 15.0% for the reverse and forward scans, respectively. We also performed the forward and reverse *J–V* scans at different rates and show that the performance is quite insensitive to the scan rates (Figure S5 and Table S4, Supporting Information).

The negligible hysteresis is mainly attributed to PFNOX, as PCBM: PS devices (shown in Figure S3, Supporting Information) exhibit large hysteresis but PCBM: PFNOX shows negligible hysteresis. The pure PCBM‐based solar devices also exhibit large hysteresis, which is due to the fast recombination by the surface trap states of perovskite films. As shown in a recent report, it is important to achieve balanced electron and hole transports in PKSCs to reduce hysteresis, our results clearly demonstrated that the electron transport ability of PCBM was a limiting factor in our PKSCs. By doping and improving the electron transport ability of the PCBM ETL, the performance of PKSCs can be dramatically enhanced. It is also reasonably to expect that our approach of improving the PCBM ETL can be combined with previous methods on improving the perovskite active layer, as faster electron transport should always be a desirable change for PKSCs.

As PFNOX has been demonstrated to dope PCBM, it is reasonable to expect that the PFNOX doped PCBM can facilitate the electron transfer from the perovskite layer to the ETL layer and thus improving the cell performance. To prove this hypothesis, steady state, and transient PL spectroscopy were employed to study the charge carriers extraction process from the perovskite absorber to the PCBM layer.^20^ The steady state PL spectra of pure halide CH_3_NH_3_PbCl_3−*x*_I*_x_* perovskite and perovskite/ETL films are shown in **Figure** **3**A. All of the samples exhibit a PL peak at 760 nm originated from the perovskite CH_3_NH_3_PbCl_3−*x*_I*_x_*. The PL intensity was largely quenched after the PCBM electron accepting layer coating. However, the extent of PL quenching is more significant for the perovskite sample with PCBM:PFNOX. The PL intensity of the PFNOX‐doped PCBM sample decreased by 21 times compared to that of the perovskite sample, while the PL intensity decrease was ≈15 times for the nondoped PCBM sample. This observation indicates that PFNOX doping enables better charge separation from perovskite absorber to the ETL.

**Figure 3:**
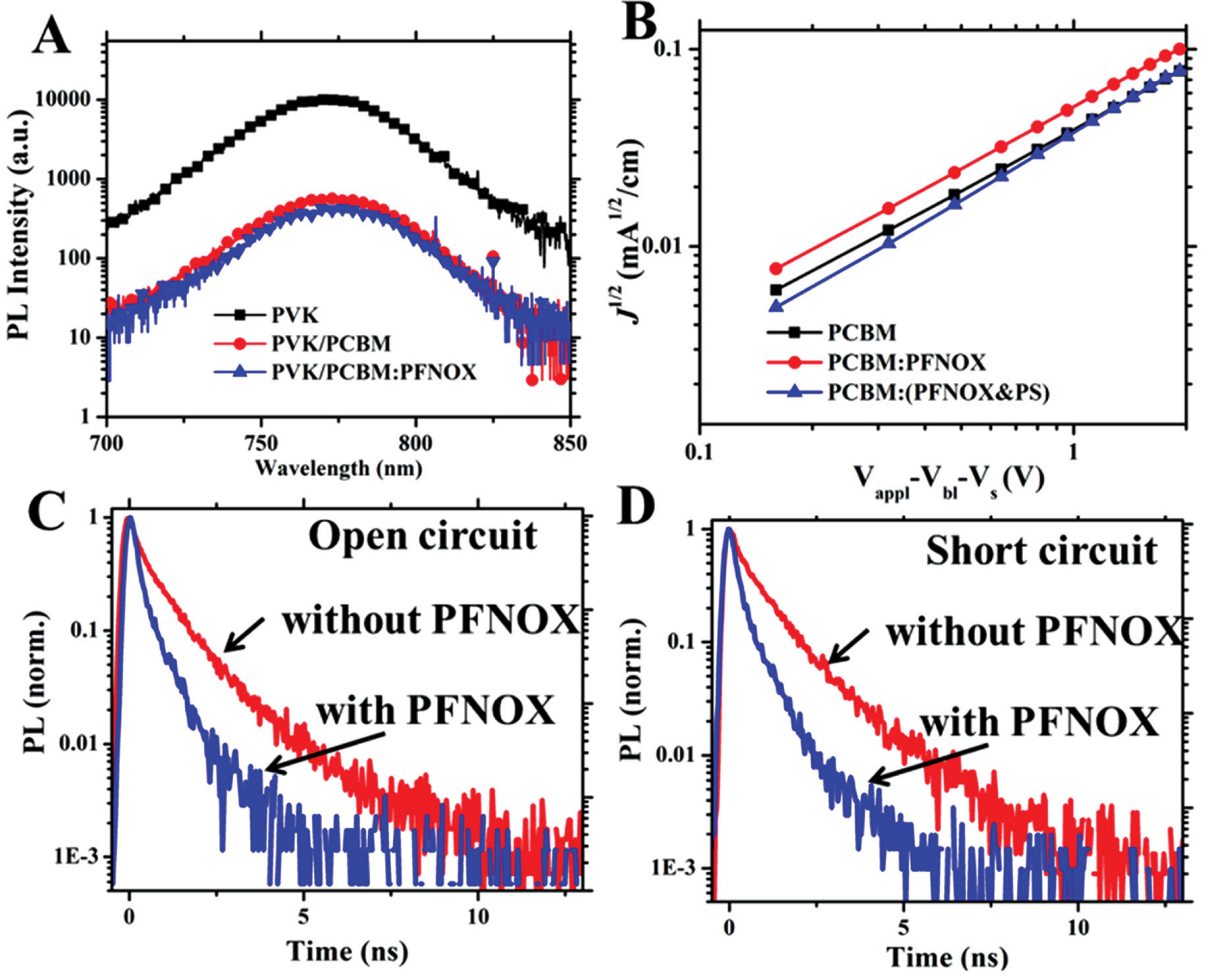
A) Steady‐state PL spectra of perovskite (PVK), PVK/PCBM, and PVK/PCBM film. B) The injection characteristics of the electron‐only devices based on the structure of ITO/ZnO/electron transport layer/Ca/Al. Normalized PL decay of the devices C) with open‐circuit and D) with short circuit. The steady‐state PL and transient PL was excited at 514.5 nm (20 mW) and 400 nm (150 fs pulse, 3.8 MHz, 31 micro‐W), respectively.

We tested the electron mobility based on electron‐only devices with the structure of ITO/ZnO/electron transport layer/Ca/Al. The *J–V* curves are shown in Figure 3B and the electrical parameters are summarized in Table S1 (Supporting Information). The result indicates that the electron transport ability of the PCBM:PFNOX is improved because PFNOX can effectively dope the PCBM ETL and thus enabling a greater electron transport ability. Also, our cyclic voltammetry and UV–vis measurements (Figures S1 and S2, Supporting Information) confirmed that the PFNOX‐doped PCBM ETL has similar energy levels to the pure PCBM ETL.

It is important to note that our study is distinctively different from previously reported hybrid perovskite/polymer solar cells,[[qv: 6f]] which used a light‐absorbing polymer:fullerene blend (≈50%:50% weight ratio) that contribute to the photo‐current generation of the integrated cell. In our approach, the two polymers only account for 1–4 wt% in weight for the blend. In addition, the PFNOX polymer and polystyrene used in our study have very weak light‐absorption properties (Figure S1, Supporting Information) in nature. They only function as low‐concentration dopants to the PCBM ETL and do not contribute to the photo‐current of the PKSCs. In this sense, the cells described in the previous report[[qv: 6f]] are truly hybrid perovskite/polymer bulk‐heterojunction (BHJ) solar cells, in which both the perovskite part and the polymer:fullerene BHJ film contribute to the photocurrent. However, our cells with PCBM:(PS&PFNOX) is still the typical inverted PKSCs that utilize a PCBM ETL with improved electron transport ability upon doping with two photo‐inactive polymers.

To examine the transfer process of free charges in these devices, we measured time‐resolved PL spectroscopy directly on these solar cell devices for PCBM and PCBM:PFNOX‐coated perovskite films. Figure 3C,D shows the decay traces of the emission peaks at 760 nm (1.63 eV) for the two devices with or without PFNOX at short‐circuit and open‐circuit conditions. More details for the measurement can be found in the Supporting Information and the fitting decay time is shown in Table S2 (Supporting Information). From these data, it is clear to conclude that the electron transfer from the perovskite to the ETL is faster for the PFNOX‐doped PCBM‐based than that for the neat PCBM‐based devices, where the electron transfer rate of the PFNOX‐based devices was ≈0.3 ns while that of the devices with neat PCBM was only ≈0.7 ns, suggesting PFNOX doping improves the electron transfer property.

To further study the charge transfer process, especially the back recombination from ETL to perovskite layer, we measured devices under operating condition by EIS, which is often used to measure charge transfer and recombination parameters in solar cells, such as chemical capacitance, recombination resistance, charge conductivity, etc.^21^, ^22^ **Figure** **4** presents Nyquist plots of the solar cells with PCBM: PFNOX (Figure 4A) and PCBM (Figure 4B) based devices, which were recorded at different applied voltages under 1‐sun illumination. The frequency range for all of the EIS measurements is from 0.5 Hz to 1 MHz. All devices were applied a bias voltage ranging from 0 to 0.9 V as transport in the high frequency region would meet very small resistance, making it difficult to model the exact transport resistance value. The Nyquist plots typically can be demarked into two main regions or arcs at high and low frequencies.

**Figure 4:**
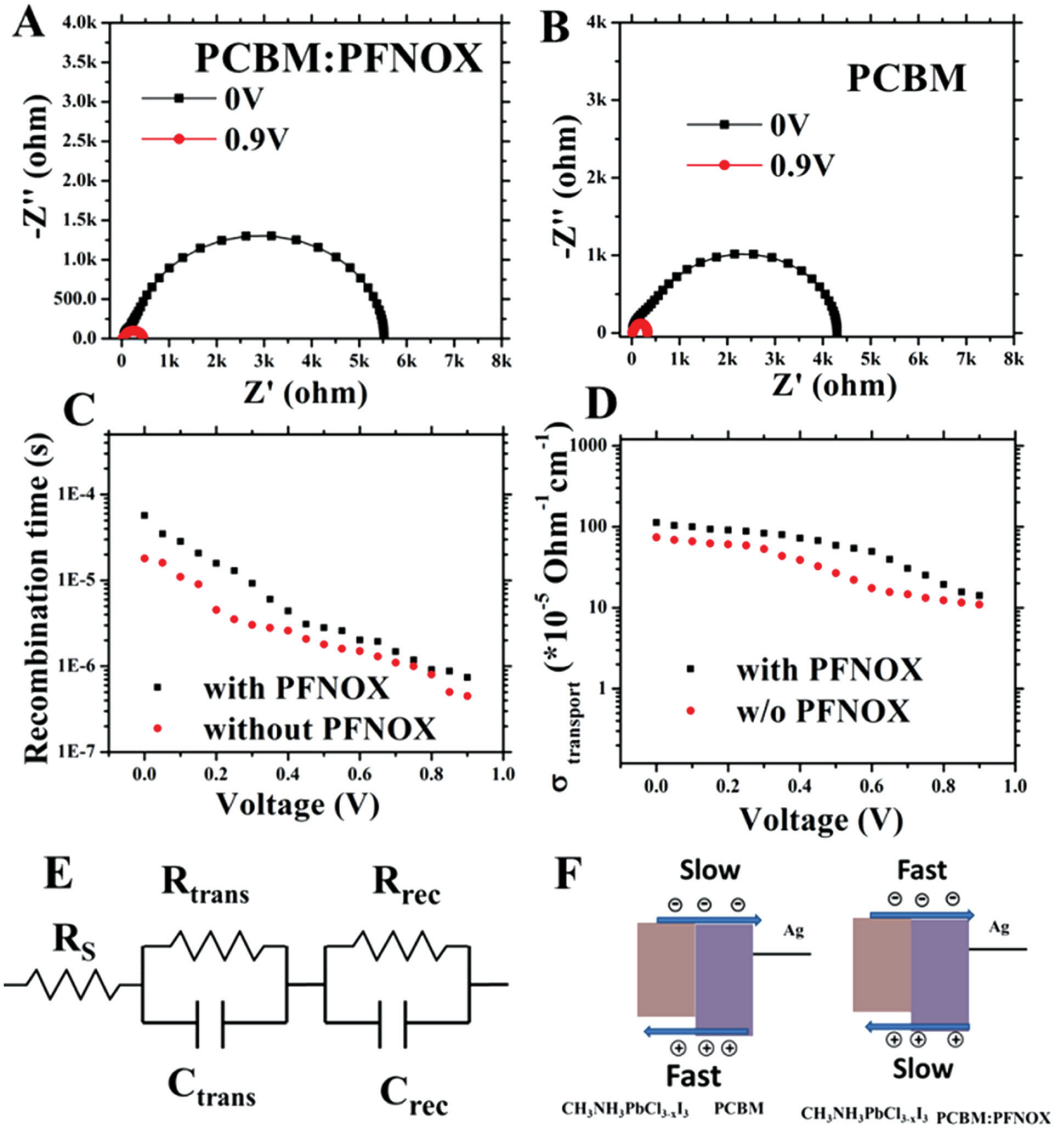
Impedance spectroscopy characterization. The representative Nyquist plots of whole regions of impedance spectra at different biases (short‐circuit 0 V and applied bias voltage 0.9 V) with PFNOX additive A) and without PFNOX additives B) into PCBM layer under AM 1.5 G illumination at 100 mW cm^−2^. Plots a series of characteristic recombination time C) and transport conductivity *σ* transport D) from impedance spectroscopy measurements obtained from the cells at different bias voltage under 1 sun illumination. The equivalent circuit E) used for fitting the Nyquist plots. F) Schematic of the charge transport at perovskite/PCBM with or without PFNOX doping.

We fitted our data based on a commonly used equivalent circuit model shown in Figure 4E. Here, in the high frequency region, a transport resistance (*R*_trans_) with parallel chemical capacitance (*C*_trans_) subcircuit was applied. Then the arc in the low frequency region is assigned to the recombination resistance *R*_rec_ and the parallel chemical capacitance *C*_rec_. We introduce a recombination lifetime (Figure 4C) which combines the surface and bulk charge recombination lifetime. Here with the PFNOX doping, the recombinaion life time is much larger, almost one order of magnitude, than without PFNOX doping devices, which proves the recombination routes from PCBM back to perovskite is suppressed. We also calculated the transport mobility from high frequencies arc of the Nyquist plots in Figure 4D. Considering that the only variable factor is PFNOX, we can conclude that the improved tranport mobility is due to the PFNOX doping, as it has been demonstrated before that PFNOX can effectively dope PCBM^12^ and the doped PCBM apparently should exhibit a better charge transport ability than the nondoped counterpart.(also supported by our electron‐only device results in Figure 3B).

In summary, we have successfully developed a new polymer:PCBM hybrid ETL that is very simple yet appears to have dramatic impacts in improving the PCE and reducing the hysteresis of PKSCs. The PFNOX‐doped devices exhibit a significant enhancement of short‐circuit photocurrent (*J*_SC_) and PCE of 14.0% compared the PCE of 11.4% for devices without PFNOX. Furthermore, a second additive (polystyrene) was introduced to further increase the efficiency to 16.2% with negligible hysteresis. While the function of the polystyrene additive is to increase the film quality of the ETL, the reduced hysteresis is mainly attributed to the electrical doping of PFNOX to PCBM. Through time‐resolved PL and EIS study, the improved PCE and reduced hysteresis are attributed to the faster electron transfer and reduced electron recombination for the PFNOX doped devices. Our work offers a simple and effective approach to addressing hysteresis problem for PKSCs. By combining our new ETL with existing approaches in the PKSC field, further improvement in PCE and hysteresis may be possible.

## Supporting information

As a service to our authors and readers, this journal provides supporting information supplied by the authors. Such materials are peer reviewed and may be re‐organized for online delivery, but are not copy‐edited or typeset. Technical support issues arising from supporting information (other than missing files) should be addressed to the authors.

Supplementary
