# Supplementary material for: A PCBM Electron Transport Layer Containing Small Amounts of Dual Polymer Additives that Enables Enhanced Perovskite Solar Cell Performance
Source: Adv Sci (Weinh). 2015 Dec 10;3(9):1500353. doi: 10.1002/advs.201500353 (PMC5039980; doi:10.1002/advs.201500353)
Supplement: Supplementary file 1 — Supplementary [file ADVS-3-0g-s001.pdf]

Copyright WILEY-VCH Verlag GmbH & Co. KGaA, 69469 Weinheim, Germany, 2015.

ADVANCED SCIENCE
Open Access

Supporting Information

for *Adv. Sci.*, DOI: 10.1002/advs. 201500353

A PCBM Electron Transport Layer Containing Small Amounts of Dual Polymer Additives that Enables Enhanced Perovskite Solar Cell Performance

Zonglong Zhu, Qifan Xue, Hexiang He, Kui Jiang, Zhicheng Hu, Yang Bai, Teng Zhang, Shuang Xiao, Kenan Gundogdu, Bhoj Raj Gautam, Harald Ade, Fei Huang, Kam Sing Wong, Hin-Lap Yip, Shihe Yang,* and He Yan**

Copyright WILEY-VCH Verlag GmbH & Co. KGaA, 69469 Weinheim, Germany, 2013.

Supporting Information

A PCBM electron transport layer containing small amounts of dual polymer additives that enables enhanced perovskite solar cell performance

Zonglong Zhu †, Qifan Xue ‡, Hexiang He ⊥, Kui Jiang †, Zhicheng Hu ‡, Yang Bai †, Teng Zhang †, Shuang Xiao †, Kenan Gundogdu §, Bhoj raj Gautam §, Harald Ade §, Fei Huang ‡, Kam Sing Wong ⊥, Hin-Lap Yip ‡, Shihe Yang †*, He Yan †**

†, Department of Chemistry, The Hong Kong University of Science and Technology, Clear Water Bay, Kowloon, Hong Kong

‡ Institute of Optoelectronic Materials and Devices, State Key Laboratory of Luminescent Materials and Devices, South China University of Technology Guangzhou, P. R. China

§ Department of Physics, North Carolina State University, Raleigh, NC, USA

⊥ Department of Physics, The Hong Kong University of Science and Technology, Clear Water Bay, Kowloon, Hong Kong

E-mail: msangusyip@scut.edu.cn, chsyang@ust.hk, hyan@ust.hk

Experimental Section

Materials synthesis and device assembly

PCBM solution was prepared by dissolving Phenyl-C61-butyric acid methyl ester (PCBM, 6 g, Nano-C) into chlorobenzene with a concentration of 20 mg mL^{-1}, and stirred in glove box under 50 ^oC overnight. Then, the solution was purified by the 200 nm filter to get the pure PCBM solution. The polystyrene (Polystyrene, Mw=12,650,000, American Polymer Standards Corp.) precursor was prepared by dissolving polystyrene into chlorobenzene to obtain a solution with 6 mg mL^{-1}, and was stirring under 60 ^oC overnight before using. The PFNOX precursor was prepared by dissolving polystyrene into chlorobenzene to obtain a solution with 5 mg mL^{-1}, and was stirring under 60 ^oC overnight before using. The PS:PFNOX:PCBM solution was prepared by mixing the pure PCBM solution with the PS and PFNOX precursor at a certain ratio （PS:PFNOX:PCBM=1:2:20 volume ratio） and stirring overnight under 60 ^oC.

CH_3NH_3I was synthesized under an ice bath for 2 hrs by reacting methylamine (CH_3NH_2, 33 wt% in ethanol from Sigma-Aldrich) with hydroiodic acid (HI, 57 wt% in water from Sigma-Aldrich). The white powders were precipitated by drying at 60 °C and washed for 3 times with diethyl ether (Sigma-Aldrich) before further dried out to be stored in nitrogen-filled glove-box. $CH_3NH_3PbI_{3-x}Cl_x$ precursor solution was prepared by dissolving 0.88M lead chloride (99.999%, Sigma-Aldrich) and 2.64 M CH_3NH_3I in anhydrous *N*,*N*-Dimethylformamide (DMF, 99.8%, Sigma-Aldrich).

PEDOT:PSS was spin-coating onto ITO at 4000 r.p.m. for 30 s. Then after heating at 100 °C for 10 min, the perovskite precursor solution was spin-coated at 3000 r.p.m. for 30 s. After drying for more than 10 mins, the as spun films were annealed over 50 mins at 100 °C in the case of $CH_3NH_3PbI_{3-x}Cl_{x.}$ Then, the PCBM electron transport layer was deposited by spin coating at 1500 r.p.m for 60 s and 2000 r.p.m for 5 s. Finally, a 100-nm-thick aluminum layer was deposited by thermal evaporation at a base pressure of $1x10^{-7}$ mbar. All the process are carried out inside glove box.

Device characterization: The morphologies of the device sample were characterized by a field emission scanning electron microscope (FE-SEM; JEOL 6700F) operated at 5 kV. The surface of device sample were characterized by an atomic force microscope (Veeco diInnova) with a Si tip. Fabricated photovoltaic cells were characterized by open circuit voltage decay measurement, current-voltage (J-V) characteristics and incident photon-to-current conversion efficiency (IPCE). Photocurrent and voltage were measured by a solar simulator (Oriel, 450 W Xe lamp, AM 1.5 global filter) equipped with an electrochemical workstation (Zanher, Zennium). the Zahner controlled intensity modulated photoresponse spectroscopy (C-IMPS) system. We measured the EIS spectra at an applied bias of Voc and a frequency range from 0.5Hz and 1MHz with AC amplitude of 10 mA under illumination of simulated solar AM1.5 global light at $100mWcm^{-2}$. Z-View Analyst software was used to model the Nyquist plots obtained from the impedance measurements. The light source was calibrated to

1 sun (100 mW/cm^2) using an opticalpower meter (Newport, model 1916-C) equipped with a Newport818P thermopile detector. The IPCE measurements were carried out with a Zahner Zennium CIMPS-PCS system established with the tunable light source (TLS). Cyclic voltammetry was carried out on a CH Instruments Electrochemical Analyzer at a scan rate 100 mV s-^{1}. The film thickness was determined by a Tencor Alpha-Step 200 surface profiler system. Charge-carrier mobilities (μ) were calculated from the J–V characteristics using the space-charge-limited current (SCLC) method with the Mott-Gurney equation for the current density J_{SCLC} expressed as $J = \frac{9\varepsilon_r \varepsilon_0 \mu V^2}{8L^3}$ □, where ε0 is the vacuum permittivity, εr is the dielectric constant of the film (εr = 3 was assumed), and L is the thickness of the active layer. For time-resolved PL measurements, a tunable Ti:sapphire femtosecond-pulsed laser was used as the excitation light source (150fs pulse, 3.8MHz, 31 micro-W), with the excitation wavelength 400 nm

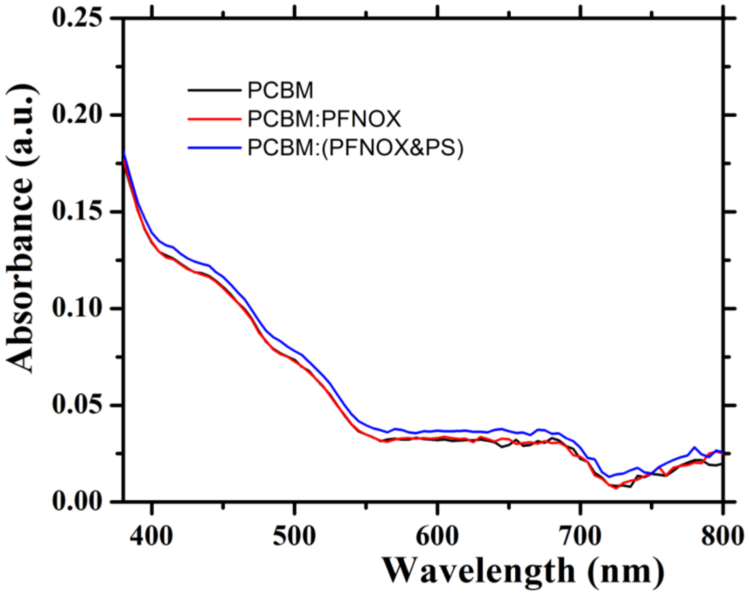

Figure S1 Absorption spectra of PCBM, PCBM: PFNOX, PCBM: (PFNOX&PS) films

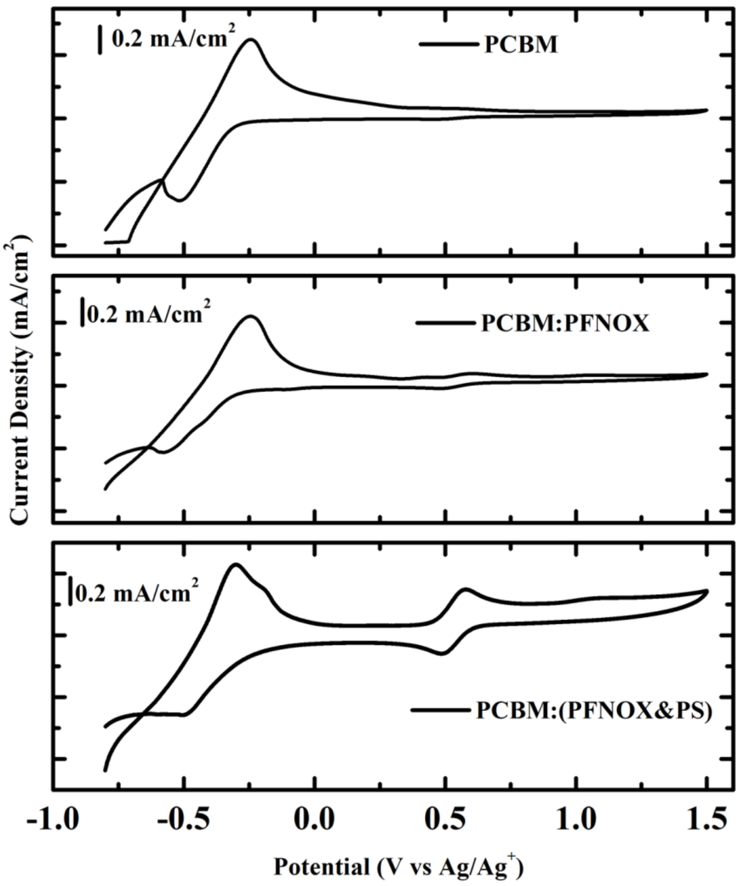

Figure S2 Cyclic voltammograms of PCBM, PCBM: PFNOX, PCBM: (PFNOX&PS) films in acetonitrile with 0.1 mol L^{-1} TBAPF6 at 100 mV s^{-1}.

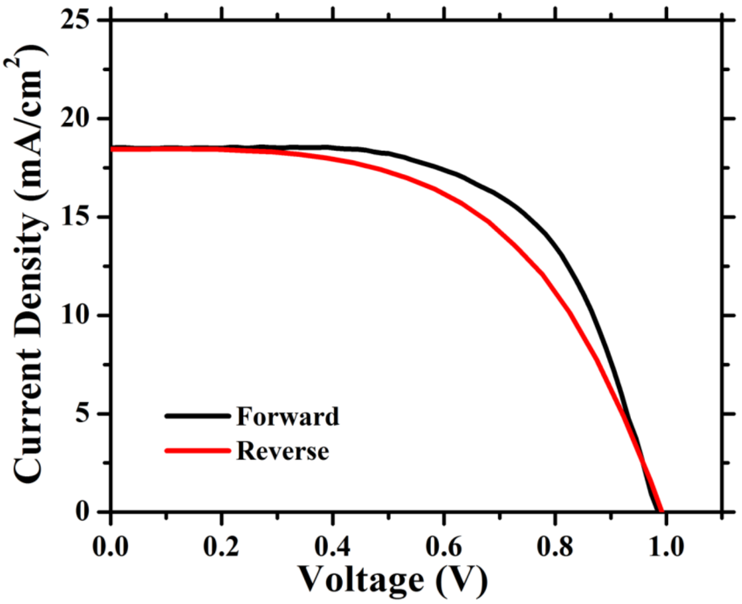

Figure S3 Photocurrent density-voltage (*J*-*V*) characteristics of PCBM: 1.5wt% PS perovskite devices, measured with 50 mV/s voltage steps.

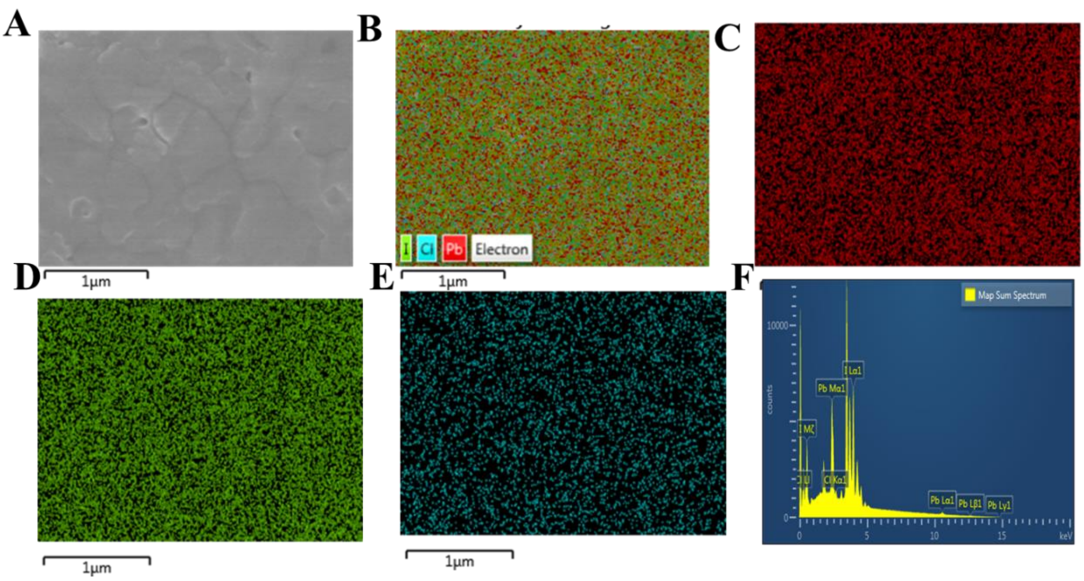

Figure S4 SEM images and EDS maps of distribution of elements on the surface of phosphogypsum (A) Top section of $MAPbCl_{3-x}I_x$ SEM image; elements distribution of overal (B), Pb(C), I(D), Cl (E), EDS analysis (F)

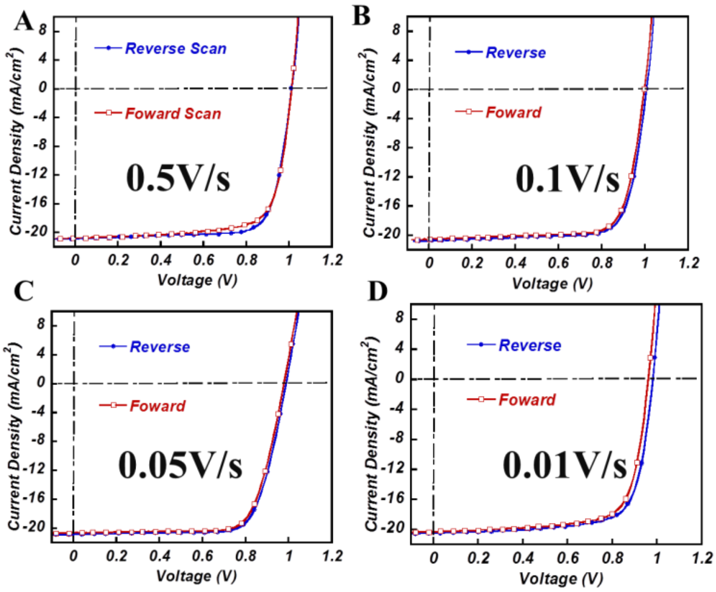

Figure S5 The solar cell performance (ITO/PEDOT:PSS/$MAPbCl_{3-x}I_x$/PCBM:PS&PFNOX/Ag) with forward and reverse scan direction at different scanning rate 0.5 V/s (A), 0.1 V/s(B), 0.05 V/s (C), 0.01 V/s (D).

Table S1: The electron mobility of the PCBM, PCBM:PFNOX and PCBM: (PFNOX&PS) films. The electron only devices for the measurements have a structure of ITO/ZnO/PCBM or composite films/LiF/Al.

	PCBM	PCBM:PFNOX	PCBM: (PFNOX&PS)
mobility/$\times 10^{-4}$ $cm^2\ V^{-1}s^{-1}$	17	28	31
Thickness (nm)	85	80	120

Table S2 Photoluminescence decay rate through open circuit and short circuit based devices of ITO/PEDOT:PSS/PVK/PCBM/Ag and ITO/PEDOT:PSS/PVK/PCBM:PFNOX/Ag

	Open circuit PL time (ns)	Short circuit PL time (ns)
ITO/PEDOT:PSS/PVK/PCBM /Ag	0.69	0.74
ITO/PEDOT:PSS/PVK/PCBM :PFNOX/Ag	0.29	0.30

Table S3 Elemental map and quantitative data of $MAPbCl_{3-x}I_x$

Element	Wt%	Wt% Sigma	Atomic %
Cl	0.31	0.09	1.31
I	61.99	0.34	71.91
Pb	37.70	0.34	26.79
Total:	100.00		100.00

Table S4 The parameters of cell performance with forward and reverse scan direction based on the structure of ITO/PEDOT:PSS/$MAPbCl_{3-x}I_x$/PCBM:PS&PFNOX/Ag at different scanning rate 0.5 V/s (A), 0.1 V/s(B), 0.05 V/s (C), 0.01 V/s (D).

**Scan Rate**	**Voc(V)**	**Jsc(mA/cm^2)**	**FF(%)**	**PCE(%)**
0.5V/s (Forward)	1.01	20.8	76.9	16.2
（Reverse）	1.01	20.8	74.5	15.7
0.1V/s (Forward)	1.01	20.8	76.3	16.0
（Reverse）	1.01	20.8	75.2	15.7
0.05V/s(Forward)	1.00	20.8	75.3	15.3
（Reverse）	1.00	20.8	76.1	15.8
0.01V/s(Forward)	0.99	20.6	74.6	15.2
（Reverse）	1.00	20.6	75.1	15.5

Table S5 Parameters of solar cells performance between the normal structure devices and inverted device

**Scan Rate**	**Voc(V)**	**Jsc(mA/cm^2)**	**FF(%)**	**PCE(%)**
Inverted structure [a] (Forward)	1.01	20.8	76.9	16.2
（Reverse）	1.01	20.8	74.5	15.7
Normal structure[b] (Forward)	1.02	20.2	70.3	14.5
（Reverse）	0.95	17.8	55.2	9.33

a. Inverted structure (ITO/PEDOT:PSS/$MAPbCl_{3-x}I_x$/PCBM:PS&PFNOX/Ag)
b. Normal structure (FTO/TiO2/ $MAPbCl_{3-x}I_x$/Spiro-OMeTAD/Ag)
